# Exploring Additional Dimensions of Complexity in Inhibitor Design for Serine β-Lactamases: Mechanistic and Intra- and Inter-molecular Chemistry Approaches

**DOI:** 10.3389/fmicb.2018.00622

**Published:** 2018-04-05

**Authors:** Focco van den Akker, Robert A. Bonomo

**Affiliations:** ^1^Department of Biochemistry, Case Western Reserve University School of Medicine, Cleveland, OH, United States; ^2^Medicine, Pharmacology, Molecular Biology and Microbiology, Proteomics and Bioinformatics, Case Western Reserve University School of Medicine, Cleveland, OH, United States; ^3^Medical Service and Geriatric Research, Education, and Clinical Centers (GRECC), Louis Stokes Cleveland Department of Veterans Affairs Medical Center, Cleveland, OH, United States; ^4^Case Western Reserve University-VA Medical Center for Antimicrobial Resistance and Epidemiology (Case VA CARES), Cleveland, OH, United States

**Keywords:** beta-lactamase, structural biology, enzyme inhibitors, transition state, antibacterial agents

## Abstract

As a bacterial resistance strategy, serine β-lactamases have evolved from cell wall synthesizing enzymes known as penicillin-binding proteins (PBP), by not only covalently binding β-lactam antibiotics but, also acquiring mechanisms of deacylating these antibiotics. This critical deacylation step leads to release of hydrolyzed and inactivated β-lactams, thereby providing resistance for the bacteria against these antibiotics targeting the cell wall. To combat β-lactamase-mediated antibiotic resistance, numerous β-lactamase inhibitors were developed that utilize various strategies to inactivate the β-lactamase. Most of these compounds are “mechanism-based” inhibitors that in some manner mimic the β-lactam substrate, having a carbonyl moiety and a negatively charged carboxyl or sulfate group. These compounds form a covalent adduct with the catalytic serine via an initial acylation step. To increase the life-time of the inhibitory covalent adduct intermediates, a remarkable array of different strategies was employed to improve inhibition potency. Such approaches include post-acylation intra- and intermolecular chemical rearrangements as well as affecting the deacylation water. These approaches transform the inhibitor design process from a 3-dimensional problem (i.e., XYZ coordinates) to one with additional dimensions of complexity as the reaction coordinate and time spent at each chemical state need to be taken into consideration. This review highlights the mechanistic intricacies of the design efforts of the β-lactamase inhibitors which so far have resulted in the development of “two generations” and 5 clinically available inhibitors.

Resistance against β-lactam antibiotics is in large part mediated by β-lactamases. The expression of β-lactamases protects the intended targets of these antibiotics, the penicillin binding proteins (PBPs), transpeptidase and carboxypeptidase enzymes critical in the synthesis of peptidoglycan and the bacterial cell wall (Nikolaidis et al., 2014). β-lactamases provide this protection as they have evolved from PBPs to recognize β-lactams, yet have also acquired a deacylation machinery to inactivate/hydrolyze β-lactam antibiotics (Fisher and Mobashery, 2009). There are four classes of β-lactamases, A–D, with Classes A, C, and D being serine β-lactamases that have PBPs as a shared common ancestor; Class B is reserved for the structurally unrelated metallo β-lactamases (Bush, 2013).

The serine β-lactamases contain key motifs or features to recognize and facilitate the β-lactam for hydrolysis: (1) a polar pocket optimized to attract the carboxyl moieties of β-lactams; (2) an oxyanion hole to attract and stabilize the carbonyl oxygen of the β-lactam ring; (3) a catalytic serine hydroxyl moiety that attacks the carbonyl carbon atom which leads to breakage of the carbonyl carbon nitrogen bond in the β-lactam; (4) conserved residues involved in a deacylation step not present in PBPs (Class A β-lactamases utilize for example a deacylation water that is primed by E166/N170 in the omega loop, a structural motif not present in PBPs). These features are complemented by an intricate hydrogen bonding network involving conserved Lys and Ser/Tyr hydroxyl moieties, in addition to a likely substrate-assisted hydrogen donation step that aids in the catalytic mechanism of some of these enzymes (e.g., Class C β-lactamases; Bulychev et al., 1997; Patera et al., 2000). These above steps have been investigated for different β-lactamases (Strynadka et al., 1992; Bulychev et al., 1997; Chen et al., 2006; Docquier and Mangani, 2016) as well as analyzed with QM/MM calculations [(Meroueh et al., 2005; Li et al., 2011; Sgrignani et al., 2014, 2016; Tripathi and Nair, 2016; Lizana and Delgado, 2017)]. To combat the β-lactamase-mediated resistance against β-lactam antibiotics, many different β-lactamase inhibitors (BLIs) were developed often using novel strategies to overcome the deacylation machinery of β-lactamases (Papp-Wallace and Bonomo, 2016). This review summarizes the remarkable breadth of inhibitor development strategies often involving additional chemical bond rearrangements post-acylation. These chemical and mechanistic strategies might also be useful for targeting other enzymes. Overall, the successful efforts in this arena have led to five β-lactamase inhibitors being approved for clinical use and others that are still in preclinical development.

## Clavulanic acid, sulbactam, and tazobactam; the “first generation”

**Figure 1:**
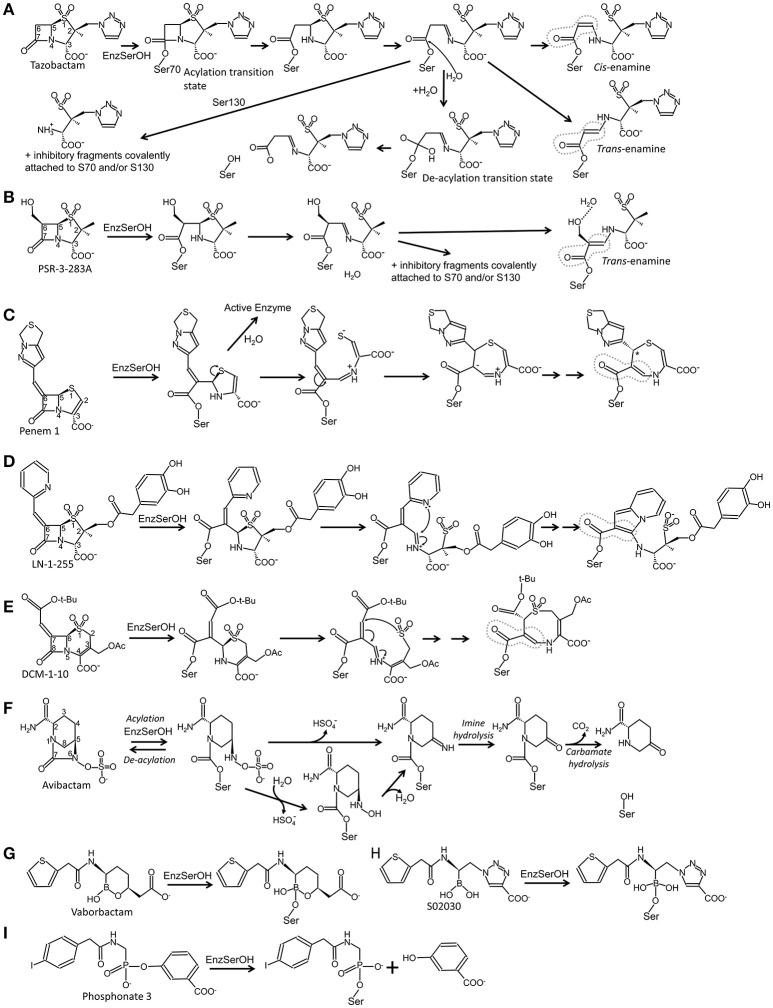
Schematic diagrams of different approaches of mechanism-based inhibition of serine β-lactamases. **(A)** Inhibition by tazobactam; **(B)** inhibition by PSR-3-283A; **(C)** inhibition by penem 1; **(D)** inhibition by LN-1-255; **(E)** inhibition by DCM-1-10; **(F)** inhibition by avibactam; **(G)** inhibition by vaborbactam; **(H)** inhibition by S02030; **(I)** inhibition by phosphonate 3. Instances where there is conjugation with the double bond of the carbonyl moiety are highlighted by a dashed gray line.

**Figure 2:**
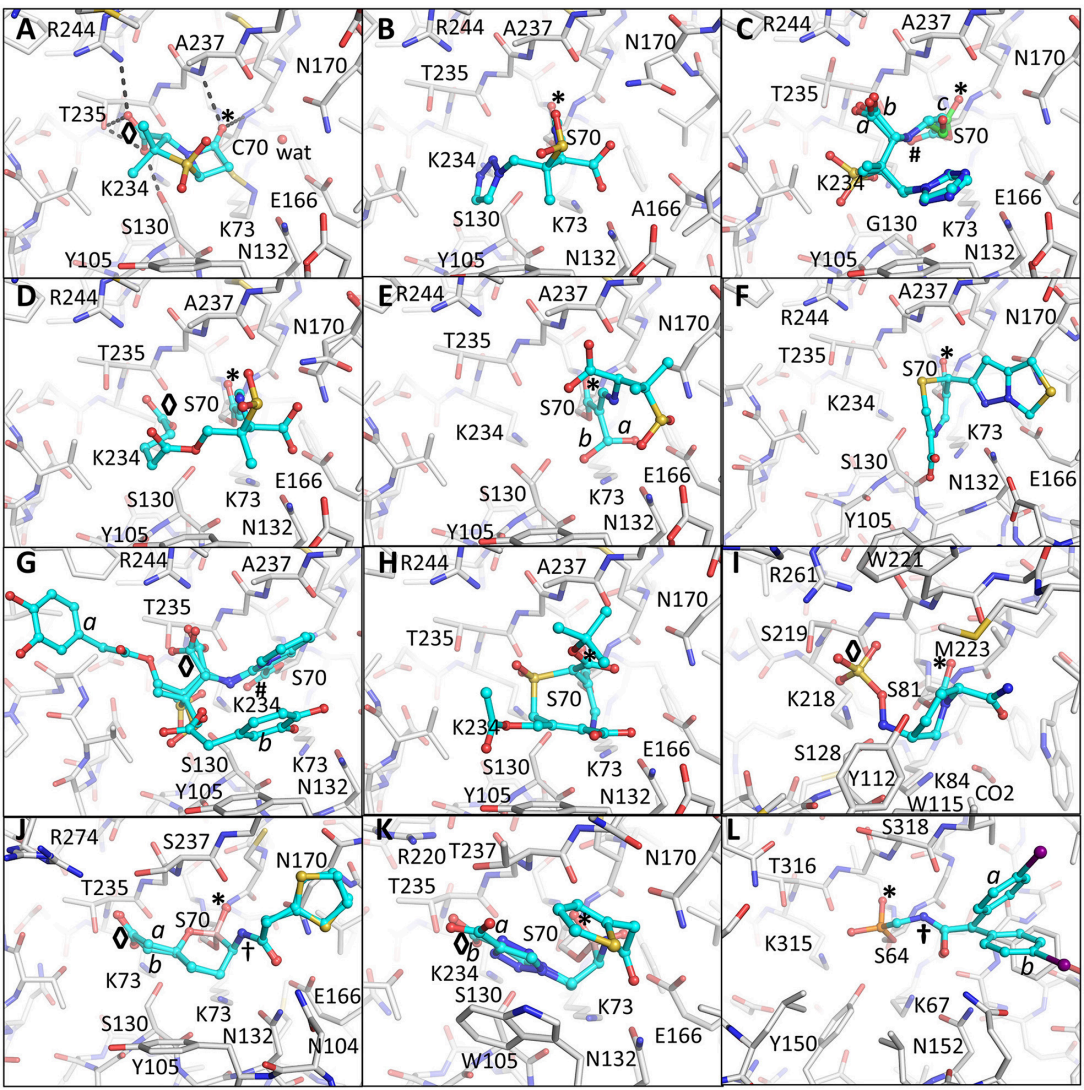
Crystallographically determined binding modes of β-lactamase inhibitors. **(A)** Sulbactam bound in a pre-acylation/Michaelis-Menten binding mode in the S70C mutant of SHV-1 β-lactamase. The S70C mutations changes the reactivity of the catalytic S70 nucleophile; the C70 residue forms a covalent sulfonamide bond with the conserved K73 allowing capture of the pre-acylation complex. Hydrogen bonds between the carboxyl and carbonyl oxygens are depicted as dashed black lines. The occupied carboxyl pocket and oxyanion hole are labeled “◇” and “*”, respectively. These labels are used through subsequent panels of this figure where applicable. The deacylation water is shown as a solid red sphere labeled “wat;” **(B)** tazobactam, in the *trans*-enamine conformation, bound to the deacylation deficient E166A mutant of SHV-1; **(C)** tazobactam, in the *cis*-enamine conformation, bound to the inhibitor-resistant S130G mutant of SHV-1. Tazobactam adopts three conformations two of which are *cis*-enamine (0.33 occupancy with cyan carbon atoms each labeled “a” and “b”) and a fragmented species with green carbon atoms labeled “c” (also 0.33 occupancy). These labels for alternate conformations are used when needed in subsequent panels of this Figure. The *cis*-enamine and fragmented species have their carbonyl oxygens positioned outside (labeled “#”) and inside the oxyanion hole (labeled “*”), respectively; **(D)** SA2-13 complexed to SHV-1; **(E)** PSR-2-283A complexed to SHV-1. The hydroxymethyl moiety was observed to be in two conformations (labeled “a” and “b”). The major conformation hydrogen bonds with the deacylation water (not shown) and the second conformation does not. Residue S130 is also in two conformations; **(F)** penem 1 bound to SHV-1; **(G)** LN-1-255 complexed to SHV-1. Two conformations of the tail of LN-1-255 are observed (“a” and “b”); **(H)** DCM-1-10 bound to SHV-1; **(I)** Avibactam bound to Class D OXA-24 β-lactamase; **(J)** Vaborbactam complexed to CTX-M-15. Two conformations for vaborbactam were observed. The amide moiety of vaborbactam (labeled “^†^”) makes hydrogen bonds across the active site groove; **(K)** S02030 bound to KPC-2. Two conformations were observed for the carboxyl-triazole moiety (labeled “a” and “b”); **(L)** Phosphonate 3 complexed to P99 β-lactamase. The iodobenzene ring was present in two conformations. Like in vaborbactam, the amide moiety of phosphonate 3 makes hydrogen bonds across the active site.

The first BLIs that were approved by the FDA were clavulanic acid, sulbactam, and tazobactam (Figure 1) (Page, 2000; Drawz and Bonomo, 2010). Each of these BLIs was paired with a β-lactam (amoxicillin/clavulanic acid, ticarcillin/clavulanic acid, ampicillin/sulbactam, cefoperazone/sulbactam, and piperacillin/tazobactam). These three inhibitors shared several features with β-lactamase substrates, such as penicillin, including a β-lactam ring fused to a 5-membered ring containing a carboxylate moiety. Sulbactam and tazobactam are penicillanic acid sulfones and differ in the C2 substituent which is a methyl group for sulbactam and a triazolyl containing moiety for tazobactam (Figure 1). In contrast, clavulanic acid is a clavam with sulfone replaced by an oxygen. The latter inhibitor differs from the other two inhibitors at the C2 position. The serine β-lactamases recognize the inhibitors by positioning the carboxyl moiety and carbonyl moieties in conserved regions in the active site as was observed in the pre-acylation Michaelis-Menten complex of sulbactam bound to the S70C mutant of SHV-1 β-lactamase (Figure 2A; Rodkey et al., 2012). The carboxyl moiety is in a pocket with hydrogen bond donors T235, S130, and within electrostatic interaction distances of R244 and K234 (Figure 2A). The carbonyl oxygen is positioned in the oxyanion hole formed by backbone nitrogens of residues 70 and 237 thereby priming the carbonyl carbon for nucleophilic attack by the hydroxyl moiety of the catalytic S70 as well as to stabilize the transition state. Finally, the hydrophobic part of the ring systems of sulbactam form hydrophobic interactions with the aromatic face of the side chain of Y105. Overall, this binding mode is similar to how these enzymes recognize β-lactam substrates (Beadle et al., 2002).

All three “first generation” inhibitors are mechanism-based compounds and inhibit serine β-lactamases by limiting deacylation *via* additional post-acylation reaction pathways that can promote semi-stable intermediates including *trans*-enamine (Figure 2B) and *cis*-enamine (Figure 2C) inhibitory species (Figure 1A; Padayatti et al., 2004, 2005; Totir et al., 2006). The enamine species both yield a double bond in the vicinity of, and thereby a conjugated system with, the carbonyl bond (Figure 1A). This conjugation is thought to decrease the carbonyl carbon's susceptibility to nucleophilic attack by the deacylation water thus preventing deacylation.

A second inhibitory mechanism for these inhibitors likely entails fashioning an eventual irreversible inhibitory species that appears after several turn-over events; this inhibitory species is postulated to involve fragmentation of the inhibitor yielding covalent modifications on either S130 and/or the catalytic S70 residue (Figures 1A, 2C; Kuzin et al., 2001; Sun et al., 2004).

## Defining the importance of a long-lived intermediate: SA2-13

Efforts to improve the longevity of the *trans*-enamine intermediate have yielded the inhibitor SA2-13 which increases the lifetime of this intermediate by 10-fold over tazobactam as observed in the measured k_*obs, react*_ (Padayatti et al., 2006). This improvement was accomplished by changing the C2 substituent to a carboxyl linker such that the latter moiety occupies the carboxyl binding pocket thereby forming a U-shaped covalent adduct that stabilizes the *trans*-enamine intermediate (Padayatti et al., 2006; Sampson et al., 2011; Ke et al., 2012c; Rodkey et al., 2014; Figure 2D). SA2-13 thus positions its two carboxyl moieties in the carboxyl binding pocket during two separate steps in its reaction with the β-lactamase. The position of the original carboxyl moiety in the SA2-13 complex leads to a minor steric and electrostatic repulsion with residues in the omega-loop such that binding of SA2-13 to Extended-Spectrum-β-Lactamase (ESBL) mutants of SHV-1 causes complete disorder of the omega loop; this disorder further enhancing SA2-13's inhibitory efficacy toward ESBLs as this loop harbors residues needed for deacylation (Sampson et al., 2011).

## Slowing the deacylation rate: 6β-hydroxymethyl containing inhibitors

The addition of a 6β-hydroxy-methyl moiety to a penicillanic acid sulfone improved certain inhibitory characteristics specifically slowing down the deacylation rate (Bitha et al., 1999a,b; Papp-Wallace et al., 2012; Che et al., 2015). The basis of this effect is attributed to the presence of the hydroxyl-methyl moiety interacting with the deacylation water when in the *trans*-enamine or imine intermediate state of the inhibitor (Figures 1B, 2E). This interaction can thereby either (1) sterically prevent the deacylation water from being able to nucleophilically attack the carbonyl carbon; and/or (2) negatively alter the nucleophilic properties of the deacylation water (Che et al., 2015). These inhibitors were also observed to undergo fragmentation yielding inhibitory adducts to Class A and C β-lactamases (Papp-Wallace et al., 2012). A 6-α-hydroxymethyl penicillanate variant yielded a similar inhibitory binding mode when bound to TEM-1 with the hydroxymethyl moiety also interacting with the deacylation water (Maveyraud et al., 1996).

## Penem; a strategic design that enhances the acyl intermediate

To take advantage of and to increase the longevity of the semi-stable acyl-enzyme imine intermediate, reactive groups were added at the C6 position such as the alkylidene group in penems (Figure 1C; Nukaga et al., 2003; Venkatesan et al., 2004a,b, 2008; Mansour et al., 2007; Ke et al., 2012b). The design was to, when in the imine intermediate, allow the nucleophilic sulfur to react with the carbon in the alkylidene group *via* a 7-*endo-trig* rearrangement. This reaction would form a new 7-membered ring that *via* additional rearrangements can lead to an enamine species that conjugates with the carbonyl carbon bond (Figure 1C). This in turn limits deacylation by decreasing the susceptibility of the carbonyl carbon to nucleophilic attack of the deacylation water. This 7-membered ring enamine intermediate was crystallographically observed for penem 1 with the carbonyl oxygen situated in the oxyanion hole with deacylation likely being diminished due to this conjugation (Figures 1C, 2F; Ke et al., 2012b).

The alkylidene moiety at the C6 position can have different aromatic 1-, 2-, or 3-ring systems as substituents of which penem 1 contains a 2-ring heterocycle substitution (Bulychev et al., 1995; Ke et al., 2012b). When combined with piperacillin, penem 1 lowered MIC values from 64–2,048 to 4–8 μg/ml for *Escherichia coli* expressing SHV-1, SHV-2, and the inhibitor-resistant R244S variant (Ke et al., 2012b).

## 6-alkylidene-2′β-substituted penam sulfones: LN-1-255 and novel chemistry

Like the penems above, alkylidene group containing reactive groups were added at the C6 position of penam sulfones (Chen et al., 1987; Buynak et al., 1999; Phillips et al., 2005; Kalp et al., 2007; Che et al., 2012). In particular, the pyridylemethylidene moiety in LN-1-255 is potent since, when in the imine intermediate, the nitrogen of the pyridyl group reacts with the carbon atom of the imine bond to form a bicyclic ring (Figure 1D; Buynak et al., 1999; Pattanaik et al., 2009). The carbonyl carbon is now conjugated with the newly formed bicyclic ring; to maintain its conjugation and thus planarity with this bulky ring, the carbonyl oxygen “flips out” of the oxyanion hole (labeled “#” in Figure 2G). This oxygen movement and the resulting conjugation renders the carbonyl bond very resistant to deacylation making the inhibitor even more efficient with a lower turn-over number compared to tazobactam (Pattanaik et al., 2009).

Remarkably, LN-1-255 and other 6-alkylidene-2′β-substituted penam sulfones are also potent Class D β-lactamase inhibitors and have a similar mechanism of enzyme inhibition (Bou et al., 2010). An additional improvement for LN-1-255 included adding a dihydroxy-phenyl catechol moiety at the C2 position of the penam sulfone. This moiety is a siderophore and could allow improved uptake of LN-1-255 *via* bacterial iron-acquisition siderophore channels (Pattanaik et al., 2009). Presently, LN-1-255 is undergoing preclinical studies to establish it efficacy in treating infections.

## 7-alkylidenecephalosporin sulfones

Like in LN-1-255 and penem 1, the alkylidene moiety can also be incorporated on the equivalent position in cephalosporin sulfones, at the 7 position (Buynak et al., 2000). Such a 7-alkylidenecaphalosporin sulfone is DCM-1-10 (Figure 1E). DCM-1-10 undergoes a similar acyl-forming inhibitory mechanism, yet deviates from penem 1 in that it is the sulfone that reacts with the carbon of the alkylidene moiety thus forming an 8 atom cyclic intermediate (Figures 1E, 2H). The carbonyl oxygen remains in the oxyanion hole yet the intermediate is likely protected from deacylation by the stabilizing effect on the carbonyl bond by being conjugated with a neighboring double bond (Figure 1E; Rodkey et al., 2013). DCM-1-10 has only modest potency as its IC_50_ is 4- and 27-fold higher for clavulanic acid and tazobactam, respectively. Nevertheless, the turnover numbers for DCM-1-10 are similar to tazobactam and the k_*obs, react*_ is significantly slower compared to tazobactam and clavulanic acid indicating that DCM-1-10 can form a relatively stable inhibitory complex (Rodkey et al., 2013).

## Diazabicyclooctane inhibitors; the “second generation”

Avibactam (NXL104) is a diazabicyclooctane (DBO) (Coleman, 2011) and is the 4th β-lactamase inhibitor that was FDA approved as part of the formulation ceftazidime/avibactam (in 2015). Unlike the above described inhibitors, avibactam inhibition of serine β-lactamases is mostly reversible (Figure 1F; Ehmann et al., 2012, 2013). Avibactam is chemically distinct from the other inhibitors in that its rings are arranged differently with the strained 4-atom β-lactam ring being absent. Nevertheless, avibactam contains a carbonyl bond adjacent to a ring nitrogen. The carboxyl moiety, present in all previously discussed inhibitors, is replaced by a negatively charged sulfate moiety. For proper recognition in the active site, the same distance between the carbonyl oxygen and the negatively charged oxygens of the sulfate group is maintained relative to its equivalent atoms in the above β-lactam containing inhibitors: the negatively charged oxygens (in either the carboxyl or sulfate moiety) and the carbonyl oxygen are separated by 4 atoms in both classes of inhibitors (Figure 1).

Avibactam forms an acyl-enzyme complex with the serine β-lactamase upon breakage of the C-N bond and concomitant opening of the 5-membered ring (Figure 1F). Interestingly, avibactam can be removed from the enzyme via deacylation and ring closure resulting in an intact avibactam molecule being liberated. A number of crystal structures have been determined of avibactam complexes with representatives from all three serine β-lactamase classes (Xu et al., 2012; Lahiri et al., 2013, 2014, 2015; King et al., 2015; Krishnan et al., 2015; Calvopiña et al., 2017; Jin et al., 2017; Lohans et al., 2017).

Despite the mostly reversible mode of inhibition (a property not evident with the other BLIs listed above), some β-lactamases are capable of slowly desulfating avibactam once bound to the enzyme resulting in inactivation of avibactam upon carbamate hydrolysis (Ehmann et al., 2012, 2013; Figure 1F). Avibactam forms similar acyl-enzyme complexes in Class A, C, and D β-lactamases (Xu et al., 2012; Lahiri et al., 2013, 2015; King et al., 2015; Krishnan et al., 2015) in which the sulfate moiety is occupying the carboxyl binding pocket and the carbonyl oxygen is situated in the oxyanion hole (Figure 2I). One possible explanation that the acyl-enzyme is likely resistant to attack by the deacylation water could be due to having a nitrogen atom bonded to the carbonyl carbon atom (Figure 1F) thereby likely altering this bond as well as its local environment. The chirality of this tertiary amine, when bound to β-lactamase, can vary from *S, R*, or planar (Krishnan et al., 2015). Additional DBO β-lactamase inhibitors are currently being developed with improved efficacies with some having dual action potential by also inhibiting PBPs (Ambrose et al., 2017; Durand-Réville et al., 2017; Moya et al., 2017a,b; Shapiro et al., 2017; Zhanel et al., 2018). The DBOs in preclinical development are listed in Table 1; additional DBO analogs in earlier stages of development can be found here (King et al., 2016; Wang et al., 2016; Durand-Réville et al., 2017).

**Table 1:** Promising DBO inhibitors in pre-clinical development or FDA approved.

**DBO name**	**Characteristics**	**References**
Avibactam (NXL104)	Currently only FDA approved DBO β-lactamase inhibitor. Partnered with ceftazidime	Reviewed in Coleman, 2011
WCK 4234	Active against *Pseudomonas* and *Acinetobacter* Class A, C, and D β-lactamases. Partnered with imipenem or meropenem	Mushtaq et al., 2017
WCK 5107 (Zidebactam)	Active against *A. baumannii, Enterobacteriaceae*, and *Pseudomonas aeruginosa*. Dual target inhibitor (*P.a*. PBP2). Partnered with cefepime or sulbactam	Livermore et al., 2017; Moya et al., 2017a,b; Sader et al., 2017a,b
WCK 5153	Active against *A. baumannii* and *P. aeruginosa*. Dual target inhibitor (*P.a*. PBP2). Partnered with cefepime or sulbactam	Moya et al., 2017a,b
ETX2514	Active against Gram-negative bacteria including *A. baumannii* and *P. aeruginosa*. Dual target inhibitor (*A. b*. PBP2). Partnered with sulbactam	Durand-Réville et al., 2017; McLeod et al., 2017; Shapiro et al., 2017
Relebactam (MK-7655)	Active against *Enterobacteriaceae, Klebsiella pneumoniae*, and *Pseudomonas*. Partnered with imipenem	Livermore et al., 2013; Blizzard et al., 2014; Lapuebla et al., 2015; Haidar et al., 2017; Lob et al., 2017
Nacubactam (OP0595)	Active against *Enterobacteriaceae, P. aeruginosa*, and *K. pneumoniae*. Dual target activity (inhibits PBP2) and has “enhancer”-activity. Partnered with cefepime, piperacillin, or meropenem	Livermore et al., 2015; Morinaka et al., 2015, 2017

## Boronic acid and phosphonate transition state analogs

Elucidation of the reaction scheme of mechanism-based inhibition BLIs (Figure 1A) suggested that transitions states can be mimicked to obtain potent inhibitors. The reaction scheme indicates that a transition state exists for both the acylation and the deacylation component of the reaction; exploiting these transitions state for developing new BLIs will be discussed next.

The cyclic boronic acid inhibitor vaborbactam (RPX7009; Hecker et al., 2015; Lomovskaya et al., 2017) was recently FDA approved (meropenem/vaborbactam) and its complex with CTX-M-15 and P99 β-lactamases was crystallographically determined (Hecker et al., 2015; Figures 1G, 2J). Vaborbactam mimics β-lactamase inhibitors/substrates (Figure 2J) by having (1) a boron atom, like the carbonyl carbon, that can be the recipient of nucleophilic attack by the catalytic serine; (2) a carboxyl moiety that occupies the carboxyl binding pocket; (3) a hydroxyl moiety mimicking the carbonyl oxygen; (4) a ring system that makes hydrophobic interactions with the Y/W side chain often found in β-lactamase active sites; and (5) an amide moiety, found in the penicillin substrate, that can interact with the different atoms across the active site cleft (with a backbone oxygen of T237 on one side of the cleft and the amide moieties of both Asn132 and Asn102 on the other end). When bound to the active site, vaborbactam adopts an acylation transition state binding mode with its exocyclic boron oxygen in the oxyanion hole (Figure 2J). Like DBOs that can reversibly acylate and deacylated, vaborbactam can be a reversible β-lactamase inhibitor (Hecker et al., 2015; Lomovskaya et al., 2017). Cyclic boronate inhibitors can have broad spectrum efficacy as some are capable of inhibiting all 4 classes of β-lactamases including metallo β-lactamases (Cahill et al., 2017). Furthermore, these inhibitors have potential beyond inhibiting β-lactamases as a cyclic boronate inhibitor was shown to inhibit PBP5 (Brem et al., 2016).

A different boronic acid inhibitor is S02030 which when complexed to KPC-2 binds in a deacylation transition state mode (Figures 1H, 2K; Nguyen et al., 2016). This is in sharp contrast to vaborbactam. S02030 possesses two boron hydroxyl groups: one of them occupies the oxyanion hole whereas the second hydroxyl occupies the pocket normally harboring the deacylation water, but this water is now displaced (Figure 2K); these hydroxyl interactions were also observed in a KPC-2 complex with a small boronic acid fragment molecule (Ke et al., 2012a). S02030 is very potent at inhibiting β-lactamases observed in *Klebsiella pneumoniae* and *E. coli* species. Developing boronic acid β-lactamase inhibitors is a promising approach as has been shown for a number of different β-lactamases (Tondi et al., 2010, 2014; Powers et al., 2014; Bouza et al., 2017; Werner et al., 2017; Caselli et al., 2018).

In addition to boronic acid analogs, phosphonates also behave as transition state BLIs. Phosphonates are unique mechanism-based BLIs; the nucleophilic attack of the catalytic serine leads to bond breakage and release of part of the molecule adjacent to the phosphonate (Figure 1I). The structure of phosphonate 3 bound to P99 β-lactamase reveals the phosphorous atom covalently bonded to the catalytic serine (Figure 2L; Lobkovsky et al., 1994). Furthermore, one of the phosphonate oxygen atoms is in the oxyanion hole and the amide moiety makes hydrogen bond interactions across the active site cleft like vaborbactam (Figures 2J, 2L). Like with boronic acids, phosphonates have also been used to probe binding modes of transition states of β-lactams such as the phosphonate transition state analog of a cephalosporin bound to a Class C β-lactamase (Nukaga et al., 2004).

## Additional inhibitor design approaches

In addition to these mechanism-based β-lactamase inhibitors, some groups have targeted developing non-covalent β-lactamase inhibitors (Eidam et al., 2012; Nichols et al., 2014). This approach is often initiated by starting from small fragments and exploiting hydrogen bond, electrostatic, and van der Waals interactions similar those observed in the mechanism-based inhibitor complexes. Despite not having a covalent bond with the catalytic serine, this approach can yield nano-molar affinity β-lactamase inhibitors (Eidam et al., 2012; Nichols et al., 2014). An important challenge here is the need to demonstrate “broad class” inhibition as was seen with DBOs. Alternatively, “narrow spectrum” inhibitors should not be discounted for therapeutic purposes as they will likely cause less damage to the patients beneficial microbiome (Boucher et al., 2017). Another approach is to utilize naturally observed protein inhibitors of β-lactamases from *Streptomyces*, termed β-lactamase inhibitor proteins (BLIPs), which can be altered to modulate β-lactamase specificity (Brown et al., 2013; Chow et al., 2016; Adamski and Palzkill, 2017a,b); peptides derived from BLIPs have been shown to have antimicrobial activity (Alaybeyoglu et al., 2015).

## Conclusion

BLI development has made tremendous progress during the last decades and exploited numerous different chemical and/or mechanistic strategies. This includes unusual (post-acylation) reactions that can involve both intra- and/or inter-molecular rearrangements. Different areas of the reaction coordinate space have been exploited to arrive at novel and promising compounds. Despite all the progress resulting in now 5 inhibitors clinically available, resistance against these β-lactamase inhibitors has occurred including against avibactam (Wright et al., 2017); resistance against vaborbactam has not been observed yet, as the inhibitor has only recently been FDA approved (Zhanel et al., 2018). Therefore, continued efforts in this field are needed to develop BLIs with novel properties such as the dual action DBO inhibitors that are in preclinical development. Also, developing BLIs that target both metallo-β-lactamases (Class B) and serine based enzymes (Classes A, C, and D) remains a goal of the future.

## Author contributions

FvdA has written the initial draft manuscript and prepared the figures. FvdA and RB have contributed to editing the manuscript.

### Conflict of interest statement

The authors declare that the research was conducted in the absence of any commercial or financial relationships that could be construed as a potential conflict of interest.
